# Agreement between a self-administered questionnaire on musculoskeletal disorders of the neck-shoulder region and a physical examination

**DOI:** 10.1186/1471-2474-9-34

**Published:** 2008-03-17

**Authors:** Nathalie Perreault, Chantal Brisson, Clermont E Dionne, Sylvie Montreuil, Laura Punnett

**Affiliations:** 1 Quebec Rehabilitation Institute, 525 Hamel Est, Quebec City, Canada; 2 Department of Social and Preventive Medicine, Laval University, Quebec City, Canada; 3 Population Health Research Unit, Laval University *Affiliated *Hospital, Quebec City, Canada; 4 Department of Rehabilitation, Laval University, Quebec City, Canada; 5 Department of Industrial Relations, Laval University, Quebec City, Canada; 6 Department of Work Environment, University of Massachusetts Lowell, Lowell, USA

## Abstract

**Background:**

In epidemiological studies on neck-shoulder disorders, physical examination by health professionals, although more expensive, is usually considered a better method of data collection than self-administered questionnaires on symptoms. However, little is known on the comparison of these two methods of data collection. The agreement between self-administered questionnaires and the physical examination on the presence of neck-shoulders disorders was assessed in the present study.

**Methods:**

This study was conducted among clerical workers using video display units. Prevalent cases were workers for whom neck-shoulder symptoms were present for at least 3 days during the previous 7 days and for whom pain intensity was greater than 50 mm on a 100 mm visual analogue scale. All 85 workers meeting this definition and a random sample of 102 workers who did not meet this definition were selected. Physical examination included measures of active range of motion and musculoskeletal strength. Cohen's kappa and global percent agreement were calculated to compare the two methods of data collection. The effect on the agreement of different question and physical examination definitions and the importance of the time interval elapsed between the administrations of the tests were also evaluated.

**Results:**

Kappa coefficients ranged from 0.19 to 0.54 depending on the definitions used to ascertain disorders. The agreement was highest when the two instruments were administered 21 days apart or less (Kappa = 0.54, global agreement = 77%). It was not substantially improved by the addition of criteria related to functional limitations or when comparisons were made with alternative physical examination definitions. Pain intensity recorded during physical examination maneuvers was an important element of the agreement between questionnaire and physical examination findings.

**Conclusion:**

These results suggest a fair to good agreement between the presence of musculoskeletal disorders ascertained by self-administered questionnaire and physical examination that may reflect differences in the constructs measured. Shorter time lags result in better agreement. Investigators should consider these results before choosing a method to measure the presence of musculoskeletal disorders in the neck-shoulder region.

## Background

Musculoskeletal disorders are among the principal causes of activity limitation and long term disability [1-3]. In 2004 musculoskeletal disorders accounted for 38% of work-related problems compensated by the Quebec Workers' Compensation Board (CSST) [4,5]. For the same year, new cases of musculoskeletal disorders (including low back pain) resulted in nearly 130 million dollars in salary compensation alone [4,5]. In the United States, musculoskeletal disorders accounted for 30% of the injuries and illnesses with days away from work in 2005 [6]. According to the US Bureau of Labor Statistics, the median length of absence resulting from musculoskeletal disorders was 9 days; among those problems, shoulders disorders resulted in the longest absences from work with a median of 15 days [6].

In epidemiological studies, data on neck-shoulder disorders are often collected by physical examination [7,8], by questionnaire [9-15] or with both instruments [16-24]. Physical examination by health professionals is usually recognized as more objective than questionnaires. However, questionnaires permit data collection on many participants for a fraction of the cost and time of a physical examination. Few epidemiological studies on neck and upper extremity musculoskeletal disorders have systematically compared the findings of questionnaires with those obtained by physical examination [16,19,22-25]. Only four studies published in English have reported the sensitivity and specificity of a questionnaire compared to clinical examination of the neck-shoulder region to identify individuals with neck-shoulder disorders [16,19,23,24].

The present study was part of a larger investigation on the prevalence of musculoskeletal disorders among video display unit (VDU) users [26]. The main objective of the present study was to assess the agreement between a self-administered questionnaire and the physical examination made by a health professional on the presence of musculoskeletal disorders of the neck-shoulder region. Secondary objectives were to assess the effects on the agreement of different questionnaire and physical examination definitions and the importance of the time interval elapsed between the administrations of the tests.

## Methods

### Study setting and selection of participants

Study participants were selected from a population of 627 women and men working in a large university and in other institutions involved in university services. To be eligible, the workers had to meet the following criteria: 1) be a clerical worker, technician, professional or executive, and 2) use a VDU for at least five hours per week. All participants had to provide an informed consent. This study was approved by the ethics committees of all the institutions involved.

Neck-shoulder disorders were defined by the presence of symptoms for at least three days during the last seven days, with peak pain intensity in the last week greater than 50 millimeters on a 100-millimeter visual analogue scale (VAS). For the agreement study, all workers meeting this definition of neck-shoulder disorders (n = 85) and a random 20% of those that did not meet this definition (n = 102) were selected. The final sample for this agreement study thus included 187 workers.

### Data collection

Arrangements had been made with the employers to allow data collection during working hours. Workers completed a self-administered questionnaire at their workstation. They were also asked to attend a physical examination at the workplace. All workers were seen between March 1994 and May 1996.

Questions related to the presence, duration and intensity of symptoms were taken from the Standardized Nordic Questionnaire [27,28] and from a standardized questionnaire used in previous studies conducted in the United States [9,29-31]. Specific questions about functional limitations were also included [28,29]. The presence of musculoskeletal symptoms during the last six months was recorded, as was the number of days where the symptoms were present during the last seven days. Workers who reported pain in the neck-shoulder region for at least three days during the last seven days, with the worst pain intensity in the last week marked above 50 millimeters on the 100-millimeter VAS [32] were considered as cases. This case definition was labeled "the primary questionnaire definition". Similar case definition based on symptom duration and pain intensity has been used in previous studies to define neck-shoulder disorders [9]. Other definitions were used in order to assess the usefulness of adding criteria related to limitations in activities of daily living (ADL). Limitations in ADL were computed as the average score of nine items rated from 1 to 5 (turn head side ways, put an object on a higher shelf, look downward, fall asleep, put on coat, drive car for more than 30 miles, lift or carry an object weighting more than ten pounds, comb hair and do usual work). Workers meeting the primary definition criteria and having an average score equal to or greater than 2 were then defined as cases for the ADL limitations definition. Other definition criteria related to limitation in work, household and leisure activities were coded as yes/no items (Table 1).

**Table 1:** Definitions and corresponding prevalence of musculoskeletal problems in the neck-shoulder region among all VDU users (n = 627)

	Prevalence (%)
**Questionnaire**	
**Primary definition : **Presence of symptoms for at least 3 days during the last 7 days, with worst pain intensity greater than 50 mm on the 100 mm VAS scale.	17.1
**Limitations in activities of daily living : **Primary definition and a score equal or greater than 2 on the functional limitations scale.	8.2
**Limitations in work activities : **Primary definition and limited work activities.*	2.9
**Limitations in household activities : **Primary definition and limited household activities.*	4.5
**Limitations in leisure activities : **Primary definition and limited leisure activities.*	5.0
**Physical examination**	
**Primary definition: any one of the following three :** • Diminution ≥ 30% of the normal active range of motion (American Academy of Orthopaedic Surgeons, 1966); • Diminution of normal muscular strength (score ≤ 4 on the Lovett scale) (Daniels, 1995); • Pain of moderate intensity or worse (≥ 3 on the 11-point Numerical Rating Scale) produced at the relevant site during any maneuver.	41.9
**Definition based solely on decreased range of motion or muscle strength : One of the following two :** • Diminution ≥ 30% of the normal active range of motion (American Academy of Orthopaedic Surgeons, 1966); • Diminution of normal muscular strength (score ≤ 4 on the Lovett scale) (Daniels, 1995).	26.2
**Definition based solely on pain manifested during maneuvers :** • Pain of moderate intensity or worse (≥ 3 on the 11-point Numerical Rating Scale) produced at the relevant site during any maneuver.	30.1

*Measured as yes/no items

The physical examination was performed according to a standard protocol used in previous studies [29,31]. At the beginning of the study, a sample of ten participants was successively examined by two occupational therapists blind to each other's scoring. Discordant results were discussed to ensure standardization of the procedures. After standardization of the procedure, the same trained occupational therapist, blind to the participants' questionnaire answers, performed all the physical examinations. The physical examination was composed of 78 items, of which 18 were related to the neck-shoulder region. The examination included measures of active range of motion (ROM) and muscular strength. Active range of motion was measured with a universal goniometer (360 degrees) and a small half-circle goniometer (180 degrees). Measured joint movements were: neck flexion, extension and lateral rotations, shoulder flexion, abduction and external rotation, wrist flexion and extension and movements of the fingers including thumb flexion and extension. Muscular strength was assessed by manual muscle testing. All these maneuvers aimed at assessing the integrity and the performance of the structures and the soft tissues surrounding joints. Decrease in ROM was considered significant if it was 30% or less of the normal expected active range of motion, based on the norms of the American Academy of Orthopaedic Surgeons [33]. Decrease in muscular strength was considered significant if it was scored 4 or less on the Lovett scale [34]. After performing each maneuver, the subject was asked to record his/her pain level on an 11-point numerical rating scale (NRS). NRS are more appropriate to use in face-to-face and telephone interviews than VAS, and their psychometric qualities are comparable to those of VAS [35,36]. Pain was considered significant if it was reported at the relevant site during maneuvers and was of moderate intensity or worse (a score of 3/10 or more).

The primary physical examination case definition identified those showing limited range of motion or decreased muscular strength or the presence of site-specific pain during maneuvers. Two other definitions were also used: one based solely on decreased range of motion or muscular strength and another based solely on pain manifested during maneuvers (see Table 1 for complete definitions).

### Analyses

Data analyses were performed with the SAS software. All workers were classified as cases or non-cases according to each of the questionnaire and physical examination definitions. The prevalence of musculoskeletal disorders in the neck-shoulder region measured according to the five questionnaire and the three physical examination definitions was estimated for the entire VDU study sample. This was done on the basis of workers who were examined (n = 187), using a weighted sum of the proportion of physical examination cases among workers negative and positive to the questionnaire.

Cohen's Kappa and global percent agreement were used as measures of agreement between results obtained from the self-administered questionnaire and the physical examination [37]. Cohen's Kappa is a measure of agreement corrected for the agreement that could be expected by chance alone [38-40]. For all Kappa values, 95% confidence intervals were calculated [37]. Landis and Koch (1977) [41] and Fleiss (1981) [37] have presented different ranges of values for Kappa according to the degree of agreement they suggest. According to them, Kappa values lower than 0.40 represent a poor agreement beyond chance, values between 0.40 and 0.75 are considered as fair to good agreement beyond chance and Kappa values higher than 0.75 represent excellent agreement beyond chance. The global percent agreement is the raw proportion of workers with the same classification on both measures [42]. Percent agreement among cases and non-cases, which corresponds to positive and negative predictive values, was also calculated, as well as sensitivity and specificity [43]. Finally, a stratified analysis was performed to determine the effect on the agreement of the time elapsed between the administrations of the questionnaire and the physical examination. The chi-square test was used to compare percentages.

## Results

The participation rate was 84% (89.2% for the cases and 77.7% for the non-cases according to the primary questionnaire definition). The VDU users in the agreement study were similar on demographic and occupational characteristics to all VDU users. Study participants were primarily female (83%). The mean age was 44 years. More than 80% of the participants were clerical workers, 11% were professional and executives and 7% were technicians. The average use of VDU was 20 hours per week.

According to the questionnaire definitions, the prevalence of musculoskeletal disorders varied from 2.9% to 17.1% (Table 1). More positive neck-shoulder findings were reported from the physical examination than from the self-administered questionnaire.

The distribution of participants according to the primary definitions (questionnaire and physical examination) and agreement values are presented in Table 2. The comparison of the primary definitions yielded a Kappa of 0.44 and a 72% global agreement. Among questionnaire cases, 79% had a positive physical examination while among non-cases, 66% were negative on examination.

**Table 2:** Distribution of study participants according to the primary questionnaire and physical examination case definitions (n = 187)

	Physical examination			
		**+**	**-**	
	
		N (expected value)	N (expected value)	
	
**Questionnaire**	**+**	67 (46)	18 (39)	85
	**-**	35 (56)	67 (46)	102
		102	85	187

Global agreement = 72%

Kappa value = 0.44, 95% C.I. = 0.31–0.56

Agreement among questionnaire cases = 79%

Agreement among questionnaire non-cases = 66%

We investigated whether different questionnaire and physical examination definitions would influence the agreement. Table 3 presents measures of agreement between the five questionnaire definitions and the primary physical examination case definition. Sensitivity and specificity are also presented. Kappa and global percent agreement obtained with the questionnaire definition that required limitations in ADL were similar to measures obtained with the primary questionnaire definition. The definition that included limitations in work activities resulted in the lowest Kappa coefficient of the study (k = 0.19). Percent agreement was always higher among cases than non-cases. Percent agreement among cases (positive predictive value) tended to increase with the inclusion of the functional limitation criteria (Table 3). For the non-cases, global percent agreement (negative predictive value) varied little, remaining around 60% for all functional limitation definitions. The inclusion of the functional criteria to the primary questionnaire definition increased specificity but decreased sensitivity figures.

**Table 3:** Agreement between the five questionnaire definitions and the primary physical examination definition

**Questionnaire definitions**	**Global agreement**			**Agreement among cases^(1)^**	**Agreement among non-cases^(2)^**	**Sn**	**Sp**
	**Kappa**	**95% CI**	**%**	**%**	**%**	**%**	**%**

1. Primary (n = 187)^(3)^	0.44	0.31–0.56	72	79	66	66	79
2. Limitations in activities of daily living (n = 153)^(3)^	0.38	0.25–0.52	69	84	64	47	91
3. Limitations in work activities (n = 128)^(3)^	0.19	0.08–0.30	63	92	59	19	99
4. Limitations in household activities (n = 135)^(3)^	0.29	0.17–0.42	66	95	61	30	99
5. Limitations in leisure activities (n = 138)^(3)^	0.27	0.14–0.40	65	83	61	31	95

Abbreviations: Sn = sensitivity; Sp = specificity

^(1) ^Cases based on questionnaire definition. Agreement among cases corresponds to the positive predictive value.

^(2) ^Non-cases based on questionnaire definition. Agreement among non-cases corresponds to the negative predictive value.

^(3) ^N of workers vary because of the requirements of each definition

When the primary questionnaire definition was compared with the three physical examination definitions, the Kappa varied from 0.30 to 0.48 (Table 4). The Kappa was lowest (0.30) when the physical definition was based only on decreased range of motion or muscular strength. The global percent agreement (66%), sensitivity (64%) and specificity (67%) were also somewhat lower with this definition. The global percent agreement tended to be similar for the physical examination definition based solely on pain manifested during maneuvers (74%) compared to the primary definition (72%). In this comparison, the Kappa values also tended to be similar (0.48 vs 0.44). Among cases, the percent agreement decreased with both alternative physical examination definitions compared to the primary definition. The definition based solely on decreased range of motion or muscular strength yielded a value for agreement among cases of 55%. Among non-cases, the percent agreement increased with both alternative definitions (75% and 82% compared to 66% for the primary physical examination definition). The percent agreement was higher among questionnaire cases compared to non-cases with the primary definition and was higher among non-cases for the two alternative definitions.

**Table 4:** Agreement between the three physical examination definitions and the primary questionnaire definition (n = 187)

**Physical Examination definitions**	**Global agreement**			**Agreement among cases^(1)^**	**Agreement among non-cases^(2)^**	**Sn**	**Sp**
	**Kappa**	**95% CI**	**%**	**%**	**%**	**%**	**%**

1. Primary Definition	0.44	0.31–0.56	72	79	66	66	79
2. Definition based on decreased range of motion or muscular strength	0.30	0.17–0.44	66	55	75	64	67
3. Definition based solely on pain	0.48	0.35–0.60	74	65	82	75	74

Abbreviations: Sn = sensitivity; Sp = specificity

^(1) ^Cases based on questionnaire definition. Agreement among cases corresponds to the positive predictive value.

^(2) ^Non-cases based on questionnaire definition. Agreement among non-cases corresponds to the negative predictive value.

Finally, we investigated whether the time elapsed between the administrations of the two tests influenced the agreement. There was an average of 38 days (range: 2 to 187) elapsed between the administrations of the questionnaire and the physical examination. A global agreement of 77% was observed for the shortest interval (21 days or less) and of 66% for the longest interval (more than 21 days apart) (Table 5). The highest Kappa value of the study (k = 0.54) was obtained when the questionnaire and the physical examination were administered 21 days or less apart. The better agreement observed with the shortest period (21 days or less) between the administrations of the two tests was reflected in both cases and non-cases, however, none of the comparisons reached statistical significance because of the limited sample size (p-values were respectively 0.10 for global agreement, 0.30 for agreement among cases and 0.31 for agreement among non-cases). For both periods, the percent agreement was higher among cases compared to non-cases. A higher sensitivity was also observed when the questionnaire and the physical examination were administered within 21 days (sensitivity = 75%) than over 21 days (sensitivity = 56%).

**Table 5:** Effect of time elapsed between the administrations of the questionnaire and the physical examination^(1) ^(n = 187)

**Time elapsed**	**Global agreement**			**Agreement among cases^(2)^**	**Agreement among non-cases ^(3)^**	**Sn**	**Sp**
	**Kappa**	**95% CI**	**%^(4)^**	**%^(4)^**	**%^(4)^**	**%**	**%**

≤ 21 days (n = 92)	0.54	0.37–0.716	77	83	71	75	80
>21 days (n = 95)	0.33	0.15–0.52	66	74	61	56	78

Abbreviations: Sn = sensitivity; Sp = specificity

^(1) ^Primary definitions were used in estimating the agreement.

^(2) ^Cases based on questionnaire definition. Agreement among cases corresponds to the positive predictive value.

^(3) ^Non-cases based on questionnaire definition. Agreement among non-cases corresponds to the negative predictive value.

^(4) ^None of the comparisons reached statistical significance (χ^2 ^test).

## Discussion

In this study of VDU users, the agreement between a self-administered questionnaire on musculoskeletal disorders of the neck-shoulder region and a physical examination of the same region was examined in a sample of university clerical workers. Prevalence figures observed with questionnaire definitions were lower than those obtained from physical examination definitions. Results show an overall Kappa of 0.44 and a global agreement of 72% between the two instruments. The agreement was not substantially improved by the addition of questionnaire criteria related to functional limitations. The agreement diminished when the physical examination definition excluded the manifestation of pain. The percent agreement tended to be higher among cases than among non-cases. Higher agreement was observed with shorter time lapses between the administrations of the tests.

In order to be valid, a measure must first be reliable [44]. The questionnaire used here was adapted from questionnaires used in previous studies [9,28-31]. Some items were taken from the Standardized Nordic Questionnaire, which showed an acceptable degree of reliability for the neck-shoulder region [27,28]. Furthermore, previous studies suggested that questions related to the presence, duration and intensity of symptoms provide reliable information on musculoskeletal symptoms [27,28,45]. Thus, it is reasonable to consider that the questionnaire used in the present study had an acceptable level of reliability.

Previous studies also provide evidence of construct validity of subjective symptoms reported in questionnaires [46]. Also, VAS are considered among the best instruments to measure pain [32]. To reduce the impact of potential error in recall in this study [44], only symptoms in the last seven days were considered. Furthermore, the fact that the questionnaire prevalence of musculoskeletal disorders in the neck-shoulder region was comparable (17%) to what was observed in previous studies on VDU workers [15,21] provides further support for the validity of outcome measures obtained from the questionnaire.

The results of the current study suggest a fair to good agreement between the presence of neck-shoulder disorders ascertained by self-administered questionnaire and physical examination. This finding is in accordance with those obtained in previous studies comparing data from questionnaire with clinical examination to identify cases of neck-shoulder disorders [16,19,23,24]. These earlier studies have concluded that self-reported neck-shoulder symptoms by questionnaire gave fairly-good to good picture of the neck-shoulders disorders prevalence.

According to previous studies, tests used in physical examination, especially measurement of range of motion and manual muscle testing, have poor to good reliability [47-53]. However, the use of a rigorous standardized protocol, pretested by the examiner at the beginning of the current study, and the fact that only one person examined all the workers favored reliability. In their literature review, Gajdosik and Bohannon (1987) concluded that there was acceptable content validity for the measurement of range of motion [47]. Nevertheless, the comparisons in the present study might have been compromised at least in part by measurement error which could explain some lack of association with symptoms.

The Kappa statistic provides a measure of agreement that corrects for the agreement that would be expected by chance alone [54]. Global percent agreement was presented as well. According to suggested classifications [37,41], all Kappa values reported in this study are relatively low. However, the Kappa statistic is strongly influenced by the prevalence of the phenomenon under study, which is determined by the observed proportion of individuals who fall in each category of the classification table. For a given observed proportion of individuals, Kappa gets its highest value when the expected proportion of positive individuals is small [55]. In this study, the expected proportions were high. This may have led to an underestimation of the true agreement beyond chance [55,56].

The different questionnaire definitions permitted the assessment of the influence of functional limitations on the agreement. The definition that included limitations in ADL gave similar agreement values when compared to the primary definition. On the other hand, definitions that included limitations in work, household and leisure activities resulted in poorer agreement. The lack of improvement in the agreement observed with the addition of functional limitations criterion may be explained by the fact that the questionnaire definition was already somewhat restrictive (pain reported in the neck-shoulder region for at least three days during the last seven days, with the worst pain intensity greater than 50 millimeters on the 100-millimeter VAS). Under these circumstances, the addition of the ADL limitations may not have contributed more information than the primary definition. Alternatively, the physical examination findings may not correspond closely enough to the domains that limit ADL. Furthermore, limitations measured in a dichotomous format (yes/no items) may not have been sufficiently sensitive in comparison to the more refined ADL limitations question. Finally, low prevalence figures (with more restrictive definitions) lead to lower Kappa values.

The inclusion of criteria related to functional limitations enhanced agreement among cases and reduced agreement among non-cases. Limitations in work and household questionnaire definitions resulted in as much as 92% and 95% agreement among cases respectively. These results suggest that the combined use of physical examination and questionnaire items that include functional limitations is useful when one wants to identify specifically cases that would be confirmed with physical examination. Results showed more workers with limitations in activities of daily living than workers with limitations in work activities. This might suggest that, in order to maintain themselves at work, workers with musculoskeletal disorders reduce their usual daily activities or they may learn to compensate in order to maintain ADL until much later in the disease process. It might also suggest that workers with musculoskeletal problems that manifested at work have already left work, due to the healthy worker effect [57]. Individuals most likely to show limitations in range of motion or in muscular strength on physical examination and to report limitations in work activities on questionnaire were thus not included in this study.

According to our results, the measure of pain intensity provoked by specific maneuvers during the physical examination offered the best agreement when compared with the self-administered questionnaire. A low agreement was obtained with the physical examination definition based solely on decrease in range of motion or muscular strength. These results are consistent with the hypothesis that musculoskeletal disorders are progressive and that patients may have symptoms before objective physical findings appear [58]. Also, cases defined by physical examination of range of motion and muscular strength may have been overlooked by the questionnaire; this would be consistent with previous studies that showed a low correlation between pain intensity and extent of tissue damaged [59,60].

The definition based on questionnaire may not measure the same concept than the physical examination. While the physical examination measures the integrity and the absolute performance of the structures and tissues, self-reported symptoms are based on actual performance and sensation, much affected by pain perception. This distinction is supported by the large impact that pain has on the agreement. The results of this study suggest that pain intensity is an important feature in the agreement between a questionnaire on musculoskeletal disorders and a physical examination and support the construct validity of a case definition based on symptoms.

The higher prevalence of findings in the physical examination than in questionnaire might be due to the selection criteria used to define non-cases according to the questionnaire. Given that the questionnaire definition was somewhat restrictive, some non-cases were not totally free of symptoms. Indeed, 26 of those 102 workers classified as non-cases according to the primary questionnaire definition had symptoms in the week prior to the questionnaire. This could have lead to a classification bias and could have attenuated the true associations with physical examination.

The time interval elapsed between the administrations of the two tests ranged from two days to six months. Better agreement (k = 0.54) was observed with a smaller time interval (21 days or less). The temporal variability present in musculoskeletal disorder symptoms and the fact that severity of pain in musculoskeletal disorders can vary from day to day depending upon the types of activities the person has engaged in [45] are inherent difficulties for the measure of agreement between two tests [46,61]. The longer interval between the tests might have allowed time for real changes in symptoms and consequently may have contributed to the relatively limited agreement found in this study. These results are consistent with those of Björkstén et al. (1999) who observed that shorter reference period for reporting musculoskeletal problems yielded better agreement between a questionnaire and a physical examination [19].

Finally, the current study's population consisted mainly of employed clerical women, thus the generalizability of the results is limited to similar populations.

## Conclusion

In conclusion, the results of this study show that the agreement between a questionnaire on musculoskeletal disorders for the neck-shoulder region and a physical examination is fair to good. Inclusion of items related to functional limitations in questionnaires appears to be of limited value to improve the agreement. It is the physical examination definition that included pain manifestations that offered the best agreement with the questionnaire. A shorter time interval between the administrations of the two tests also yields a better agreement. Investigators should consider these results before choosing a method to measure the presence of musculoskeletal disorders of the neck-shoulder region.

## Competing interests

The author(s) declare that they have no competing interests.

## Authors' contributions

This work was conducted as part of the Master's thesis of NP, under the supervision of CB and CED. NP, CB, CED, SM and LP contributed to the conception and design of the research, analysis and interpretation of data, as well as to writing the article. Data collection was conducted under the direction of CB, with participation of NP. All authors read and approved the final manuscript.

## Pre-publication history

The pre-publication history for this paper can be accessed here:



## References

[B1] Badley EM, Webster GK, Rasooly I (1995). The impact of musculoskeletal disorders in the population: are they just aches and pains? Findings from the 1990 Ontario Health Survey. J Rheumatol.

[B2] Daveluy C, Pica L, Audet N, Courtemanche R, Lapointe F (2000). Enquête sociale et de santé 1998, 2e édition.

[B3] Woolf AD, Pfleger B (2003). Burden of major musculoskeletal conditions. Bull World Health Organ.

[B4] CSST (2005). Statistiques sur les affections vertébrales 2001-2004..

[B5] CSST (2005). Statistiques sur les lésions en "ITE" du système musculo-squelettique 2001-2004..

[B6] U.S. Department of Labor (2006). Nonfatal occupational injuries and illnesses requiring days away from work in 2005.

[B7] Ranney D, Wells R, Moore A (1995). Upper limb musculoskeletal disorders in highly repetitive industries: precise anatomical physical findings. Ergonomics.

[B8] Toomingas A, Nemeth G, Alfredsson L (1995). Self-administered examination versus conventional medical examination of the musculoskeletal system in the neck, shoulders, and upper limbs. The Stockholm MUSIC I Study Group. J Clin Epidemiol.

[B9] Bernard B, Sauter S, Fine L, Petersen M, Hales T (1994). Job task and psychosocial risk factors for work-related musculoskeletal disorders among newspaper employees. Scand J Work Environ Health.

[B10] Ariens GA, Bongers PM, Hoogendoorn WE, Houtman IL, van der Wal G, van Mechelen W (2001). High quantitative job demands and low coworker support as risk factors for neck pain: results of a prospective cohort study. Spine.

[B11] Feveile H, Jensen C, Burr H (2002). Risk factors for neck-shoulder and wrist-hand symptoms in a 5-year follow-up study of 3,990 employees in Denmark. Int Arch Occup Environ Health.

[B12] Hannan LM, Monteilh CP, Gerr F, Kleinbaum DG, Marcus M (2005). Job strain and risk of musculoskeletal symptoms among a prospective cohort of occupational computer users. Scand J Work Environ Health.

[B13] Leroyer A, Edme JL, Vaxevanoglou X, Buisset C, Laurent P, Desobry P, Frimat P (2006). Neck, shoulder, and hand and wrist pain among administrative employees: relation to work-time organization and psychosocial factors at work. J Occup Environ Med.

[B14] Ostergren PO, Hanson BS, Balogh I, Ektor-Andersen J, Isacsson A, Orbaek P, Winkel J, Isacsson SO (2005). Incidence of shoulder and neck pain in a working population: effect modification between mechanical and psychosocial exposures at work? Results from a one year follow up of the Malmo shoulder and neck study cohort. J Epidemiol Community Health.

[B15] Rossignol AM, Morse EP, Summers VM, Pagnotto LD (1987). Video display terminal use and reported health symptoms among Massachusetts clerical workers. Journal of Occupational Medicine.

[B16] Akesson I, Johnsson B, Rylander L, Moritz U, Skerfving S (1999). Musculoskeletal disorders among female dental personnel--clinical examination and a 5-year follow-up study of symptoms. Int Arch Occup Environ Health.

[B17] Andersen JHK (2003). Risk factors in the onset of neck/shoulder pain in a prospective study of workers in industrial and service companies. Occup Environ Med.

[B18] Bergqvist U, Wolgast E, Nilsson B, Voss M (1995). Musculoskeletal disorders among visual display terminal workers: individual, ergonomic, and work organizational factors. Ergonomics.

[B19] Bjorksten MG, Boquist B, Talback M, Edling C (1999). The validity of reported musculoskeletal problems. A study of questionnaire answers in relation to diagnosed disorders and perception of pain. Appl Ergon.

[B20] Gerr F, Marcus M, Ensor C, Kleinbaum D, Cohen S, Edwards A, Gentry E, Ortiz DJ, Monteilh C (2002). A prospective study of computer users: I. Study design and incidence of musculoskeletal symptoms and disorders. Am J Ind Med.

[B21] Hales TR, Sauter SL, Peterson MR, Fine LJ, Putz-Anderson V, Schleifer LR, Ochs TT, Bernard BP (1994). Musculoskeletal disorders among visual display terminal users in a telecommunications company. Ergonomics.

[B22] Juul-Kristensen B, Kadefors R, Hansen K, Bystrom P, Sandsjo L, Sjogaard G (2006). Clinical signs and physical function in neck and upper extremities among elderly female computer users: the NEW study. Eur J Appl Physiol.

[B23] Kaergaard A, Andersen JH, Rasmussen K, Mikkelsen S (2000). Identification of neck-shoulder disorders in a 1 year follow-up study. Validation Of a questionnaire-based method. Pain.

[B24] Ohlsson K, Attewell RG, Johnsson B, Ahlm A, Skerfving S (1994). An assessment of neck and upper extremity disorders by questionnaire and clinical examination. Ergonomics.

[B25] Laubli T, Thomas C, Hinnen U, Hunting W, Zeier H, Mion H (1991). Evaluation of Musculoskeletal Disorders by Questionnaire. Sozial-Und Praventivmedizin.

[B26] Brisson C, Montreuil S, Punnett L (1999). Effects of an ergonomic training program among video display unit workers. Scand J Work Environ Health.

[B27] Dickinson CE, Campion K, Foster AF, Newman SJ, O'Rourke AMT, Thomas PG (1992). Questionnaire development: an examination of the Nordic musculoskeletal questionnaire. Applied Ergonomics.

[B28] Kuorinka I, Jonsson B, Kilbom A, Vinterberg H, Biering-Sorensen F, Andersson G, Jorgensen K (1987). Standardised Nordic questionnaire for the analysis of musculoskeletal symptoms. Applied Ergonomics.

[B29] Punnett L, Fine LJ, Keyserling WM, Herrin GD, Chaffin DB (1991). Back disorders and nonneutral trunk postures of automobile assembly workers. Scand J Work Environ Health.

[B30] Silverstein BA, Fine LJ, Armstrong JTJ (1987). Occupational factors and carpal tunnel syndrome. Am J Industr Med.

[B31] Silverstein BA, Fine LJ, Armstrong TJ (1986). Hand wrist cumulative trauma disorders in industry. Br J Ind Med.

[B32] Scott J, Huskisson EC (1976). Graphic Representation of Pain. Pain.

[B33] American Academy of Orthopaedic Surgeons, British Orthopaedic Association (1966). Joint motion : method of measuring and recording.

[B34] Daniels L, Worthingham C (1995). Le bilan musculaire: Technique de l'examen clinique.

[B35] Jensen MP, Turner JA, Romano JM (1994). What is the maximum number of levels needed in pain intensity measurement?. Pain.

[B36] Strong J, Ashton R, Chant D (1991). Pain intensity measurement in chronic low back pain. Clin J Pain.

[B37] Fleiss JL (1981). Statistical methods for rates and proportion.

[B38] Armstrong BK, White E, Saracci R (1992). Principles of exposure measurement in epidemiology.

[B39] Bernard PM, Lapointe C (1995). Mesures statistiques en épidémiologie.

[B40] Maclure M, Willett WC (1987). Misinterpretation and misuse of the kappa statistic. J Epidemiol.

[B41] Landis JR, Koch GG (1977). The measurement of observer agreement for categorical data. Biometrics.

[B42] Altman DG (1991). Practical statistics for medical research.

[B43] Rothman KJ, Greenland S (1998). Modern epidemiology.

[B44] Abramson JH (1984). Survey methods in community medicine : an introduction to epidemiological and evaluative studies.

[B45] Baron S, Hales T, Hurrell J (1996). Evaluation of symptom surveys for occupational musculoskeletal disorders. Am J Ind Med.

[B46] Schierhout GH, Myers JE (1996). Is self-reported pain an appropriate outcome measure in ergonomic-epidemiologic studies of work-related musculoskeletal disorders?. Am J Indust Med.

[B47] Gajdosik RL, Bohannon RW (1987). Clinical Measurement of Range of Motion - Review of Goniometry Emphasizing Reliability and Validity. Physical Therapy.

[B48] Hoppenbrouwers M, Eckhardt MM, Verkerk K, Verhagen A (2006). Reproducibility of the measurement of active and passive cervical range of motion. J Manipulative Physiol Ther.

[B49] Jepsen JR, Laursen LH, Larsen AI, Hagert CG (2004). Manual strength testing in 14 upper limb muscles - A study of inter-rater reliability. Acta Orthopaedica Scandinavica.

[B50] Marx RG, Bombardier C, Wright JG (1999). What do we know about the reliability and validity of physical examination tests used to examine the upper extremity?. Journal of Hand Surgery-American Volume.

[B51] Salerno DF, Franzblau A, Werner RA, Chung KC, Schultz JS, Becker MP, Armstrong TJ (2000). Reliability of physical examination of the upper extremity among keyboard operators. American Journal of Industrial Medicine.

[B52] Valentine RE, Lewis JS (2006). Intraobserver reliability of 4 physiologic movements of the shoulder in subjects with and without symptoms. Archives of Physical Medicine and Rehabilitation.

[B53] Viikari-Juntura E (1987). Interexaminer reliability of observations in physical examinations of the neck. Phys Ther.

[B54] Cohen JA (1960). A coefficient of agreement for nominal scales.. Educational and Psychological Measurment.

[B55] Feinstein AR, Cicchetti DV (1990). High agreement but low kappa: I. the problems of two paradoxes. J Clin Epidemiol.

[B56] Byrt T, Bishop J, Carlin JB (1993). Bias, Prevalence and Kappa. Journal of Clinical Epidemiology.

[B57] Punnett L (1996). Adjusting for the healthy worker selection effect in cross-sectional studies. Int J Epidemiol.

[B58] Stock SR (1991). Workplace ergonomic factors and the development of musculoskeletal disorders of the neck and upper limbs: a meta-analysis. American Journal of Industrial Medicine.

[B59] Gerr F, Letz R, Landrigan PJ (1991). Upper-extremity musculoskeletal disorders of occupational origin. Annu Rev Public Health.

[B60] McDowell I, Newell C (1987). Measuring health: A guide to rating scales and questionnaires.

[B61] Croft P, Pope D, Zonca M, Oneill T, Silman A (1994). Measurement of Shoulder Related Disability - Results of a Validation-Study. Annals of the Rheumatic Diseases.

